# Post-treatment skin reactions reported by cancer patients differ by race, not by treatment or expectations

**DOI:** 10.1038/sj.bjc.6603842

**Published:** 2007-06-12

**Authors:** J L Ryan, C Bole, J T Hickok, C Figueroa-Moseley, L Colman, R C Khanna, A P Pentland, G R Morrow

**Affiliations:** 1 Department of Dermatology, University of Rochester School of Medicine & Dentistry, James P Wilmot Cancer Center, Rochester, NY 14642, USA; 2 Department of Radiation Oncology, University of Rochester School of Medicine & Dentistry, James P Wilmot Cancer Center, Rochester, NY 14642, USA; 3 Northwest Community Clinical Oncology Program, Tacoma, WA 98405, USA; 4 Columbus Community Clinical Oncology Program, Columbus, OH 43215, USA

**Keywords:** treatment, skin reactions, race, expectations, self-report

## Abstract

Cancer patients may experience skin problems while undergoing chemotherapy and radiation therapy. Frequency of skin reactions may be influenced by skin pigmentation and psychological factors. A Symptom Inventory completed by 656 cancer patients nationwide before and after chemotherapy, radiation therapy, or chemotherapy plus radiation therapy was analysed to determine if treatment type, race (Black *vs* White), and pretreatment expectations influenced post-treatment skin reactions. Subsequent analysis of a local Symptom Inventory completed weekly for 5 weeks by 308 patients receiving radiation therapy examined severity of reported skin reactions. Significantly more patients receiving radiation therapy had stronger expectations of skin problems (62%) than patients receiving chemotherapy (40%, *P*=0.001) or chemotherapy plus radiation therapy (45%, *P*=0.003). Overall, expectations did not correlate with patient reported post-treatment skin problems in white (*r*=0.014, *P*=0.781) or black (*r*=0.021, *P*=0.936) patients. Although no significant difference was found between black and white patients in their pretreatment expectations of skin problems (*P*=0.32), black patients (10 out of 18, 56%) reported more skin problems than white patients (90 out of 393, 23%, *P*=0.001). Similarly, the local study showed that significantly more black patients (1 out of 5, 20%) reported severe skin reactions at the treatment site than white patients (12 out of 161, 8%). A direct correlation was observed between severity of skin problems and pain at the treatment site (*r*=0.541, *P*<0.001). Total radiation exposure did not significantly correlate with the report of skin problems at the treatment site for white or black patients. Overall, black patients reported more severe post-treatment skin problems than white patients. Our results suggest that symptom management for post-treatment skin reactions in cancer patients receiving radiation treatment could differ depending on their racial background.

Dermatitis and other skin ailments affect 30–40% of individuals in the general population (Freak, 2004). In the US and European populations, approximately 2% of individuals suffer from psoriasis (Freak, 2004; Gelfand *et al*, 2005). Skin problems are among the most common side effects of cancer treatment reported by patients, especially those undergoing radiation therapy for breast cancer and head and neck cancer. The combination of chemotherapy and radiation therapy has previously been reported to cause the worst skin reactions because chemotherapeutic drugs can induce radiosensitivity (Alley *et al*, 2002). The role that treatment regimen, skin pigmentation, and psychological factors play in the frequency of skin reactions experienced by cancer patients remains unclear. Possible prognostic factors for cancer treatment-related skin reactions should be identifiable by investigating the skin problems reported by cancer patients of different races in relation to their treatment type and pretreatment expectations.

Skin pigmentation depends on the amount and distribution of a ubiquitous pigment known as melanin (Nielsen *et al*, 2006). The skin of darkly pigmented individuals contains larger amounts of melanin, present as granules in melanocytes, resulting in a darker skin tone. Melanin protects human skin from ultraviolet (UV) and ionising radiation damage through its capacity to absorb light over a broad spectrum (Nielsen *et al*, 2006). Despite the protective capacity of melanin, both UV and ionising radiation can cause irreparable skin damage. Consequently, cancer patients have more severe radiation-induced skin reactions in sun-exposed areas of the skin, suggesting additive damage (Johansson *et al*, 2002).

Psychological factors may also contribute to the toxicity experienced by cancer patients undergoing treatment. The range and severity of treatment side effects among cancer patients is inadequately explained by pharmacological data (Koo and Lebwohl, 2001). Schreier and Williams (2004) demonstrated that high anxiety at the start of treatment was associated with decreased quality of life in women undergoing radiation therapy or chemotherapy for breast cancer. Current data suggest that further research is required to completely understand the impact that psychological factors, such as expectations, have on the cancer treatment process. The focus of this study is to examine the influence of expectations, race, and treatment type on post-treatment skin reactions reported by cancer patients.

This correlative study of two Symptom Inventories completed by cancer patients nationwide (*n*=656) and locally (*n*=308) begins to establish the relationship between race (Black *vs* White), treatment regimen (chemotherapy and/or radiation therapy), and/or pretreatment expectations with post-treatment skin reaction as reported by cancer patients. We hypothesised that black patients would have less frequent and less severe skin problems because the high melanin content of their skin would shield skin from superficial damage. Cancer treatment with radiation therapy requires repeated exposures to low-dose ionising radiation for 6 weeks, on average. Skin pigmentation is highly influenced by radiation therapy, even when small amounts of melanin (i.e., pigmentation) are present.

## MATERIALS AND METHODS

### Nationwide Symptom Inventory

The nationwide Symptom Inventory, a single-page questionnaire, was adapted from a similar measure created at MD Anderson Cancer Center (Edwards *et al*, 2001b; Riley *et al*, 2002). The Symptom Inventory has been used by our group in several studies involving patients with cancer. The nationwide Symptom Inventory consisted of a series of visual analog scales assessing the severity of 12 symptoms (pain, fatigue, nausea, sleep problems, feelings of depression, shortness of breath, difficulty remembering things, weight loss, hair loss, difficulty concentrating, hot flashes and skin problems). Patients are asked to indicate the severity of each symptom at its worst ‘in the past week’ by filling in the appropriate circle on an 11-point horizontal scale anchored by 0=‘not present’ and 10=‘as bad as you can imagine.’ The presence of a symptom was defined as a score of at least ‘1’ on the 11-point Symptom Inventory scale. The present study focused on the presence and severity of skin problems in relation to the treatment regimen and race.

This report analysed 656 cancer patients who participated in the nationwide Symptom Inventory study conducted at 31 private community practice sites. All of the patients were approached and asked to complete a Symptom Inventory by a clinical research associate. Completion of the questionnaire was voluntary and took approximately 5 min. The cancer patients completed the Symptom Inventory before and after receiving chemotherapy, radiation therapy, or both types of treatment. The completed Symptom Inventories became part of the patient's clinical chart, and the forms could be reviewed by clinical staff during regularly scheduled patient follow-up visits. All patients included in this study signed a patient authorisation form allowing the information obtained from the Symptom Inventory to be stored in a Symptom Inventory database that is maintained by the James P Wilmot Cancer Center Behavioral Medicine Unit for research purposes. Permission to maintain the database and for patient chart review has been obtained from the University of Rochester Research Subjects Review Board in accordance with HIPAA policies.

### Local Symptom Inventory

The local Symptom Inventory, a single-page questionnaire, was also adapted from a similar measure created at MD Anderson Cancer Center (Edwards *et al*, 2001b; Riley *et al*, 2002). The local Symptom Inventory consisted of a series of visual analog scales, identical to those on the nationwide Symptom Inventory, assessing the presence and severity of 13 symptoms (pain in treatment site, other pain, nausea, vomiting, feeling distressed or upset, shortness of breath, difficulty remembering things, lack of appetite, diarrhoea, urination change, skin problems in treatment site, disturbed sleep, and fatigue). This study focused on the presence and severity of skin problems and pain in treatment site in relation to total radiation exposure and race.

This report analysed 308 cancer patients who participated in a local Symptom Inventory conducted at the James P Wilmot Cancer Center (JPWCC). Similar to the nationwide Symptom Inventory, completion of the local Symptom Inventory was voluntary and took approximately 5 min. The cancer patients completed the questionnaire before and after receiving radiation therapy. The completed Symptom Inventories became part of the patient's clinical chart and the forms could be reviewed by clinical staff during regularly scheduled patient follow-up visits. All patients included in this study signed a patient authorisation form allowing the information obtained from the Symptom Inventory to be stored in a Symptom Inventory database that is maintained by the James P Wilmot Cancer Center Behavioral Medicine Unit for research purposes. Permission to maintain the database and for patient chart review has been obtained from the University of Rochester Research Subjects Review Board in accordance with HIPAA policies.

### Statistical analyses

The Statistical Package for Social Sciences (SPSS 12.0) was used for all data processing. Trends and distributions were examined using histograms and scatterplots. Descriptive statistics were performed using non-parametric tests (Kruskal Wallis and Mann–Whitney), median test, independent *t*-tests, bivariate correlation (Pearson), and linear regression analyses. Correlative analyses were performed using Pearson's correlation. Sigma Plot 9.0 was used for graphical design.

## RESULTS

All of the patients in both subsets had normal skin before cancer treatment (severity⩽2) and reported their race as ‘White’ or ‘Black’ on the nationwide Symptom Inventory (*n*=656) and the local Symptom Inventory (*n*=308). The demographics of the cancer patients who completed the nationwide and local Symptom Inventory can be seen in Table 1. The patients were equally distributed in age groups <61 years of age and ⩾61 years of age for both questionnaires. The majority of the patients who completed the nationwide Symptom Inventory were female (65%) and reported their race as ‘White’ (95%). The nationwide survey included patients diagnosed with the following types of cancer: haematologic, head and neck, lung, gastrointestinal, genitourinary, gynaecologic, breast, or unknown primary (Table 2). The majority of patients were diagnosed with breast (51.5%), genitourinary (19.2%), lung (6.8%), or haematologic (6.3%) cancers. For the local Symptom Inventory, the proportion of female patients (54%) was similar to the proportion of male patients (46%), and the majority of the patients reported their race as ‘White’ (97%). The local survey included patients diagnosed with the following types of cancer: haematologic, brain and peripheral nervous system (PNS), head and neck, lung, gastrointestinal, genitourinary, gynaecologic, breast, skin, melanoma, soft tissue sarcoma, bone/cartilage, or unknown primary (Table 2). The majority of patients were diagnosed with breast (36.7%), genitourinary (19.2%), brain and PNS (12.7%), or lung (10.4%) cancers.

Of the 656 cancer patients who completed the nationwide questionnaire, 245 (37%) patients reported no post-treatment skin reaction (severity=0) and 411 (63%) patients reported a post-treatment skin reaction (severity⩾1). Of the 308 cancer patients who completed the local questionnaire, 142 (46%) patients reported no post-treatment skin reaction (severity=0), and 166 (54%) patients reported a post-treatment skin reaction (severity⩾1). In both patient groups, more post-treatment skin reactions were reported by patients diagnosed with breast or lung cancer who were in the younger age group (<61 years), female, and White (Tables 1 and 2).

### Reported severity of skin reactions did not differ between cancer treatments

**Figure 1:**
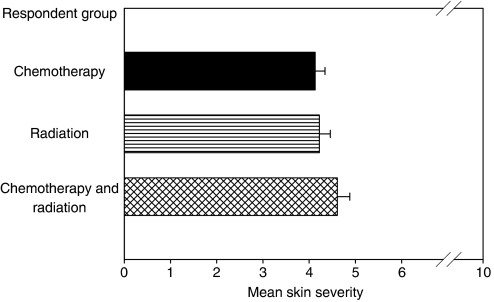
The severity of skin reactions reported by cancer patients is not influenced by treatment type. The mean and standard deviations (s.d.'s) of reported skin problems had minimal variation across treatment groups: chemotherapy (mean=4.17, s.d.=2.7); radiation (mean=4.2, s.d.=2.8); chemotherapy and radiation (mean=4.6, s.d.=2.9). No statistically significant difference was observed (*P*=0.586).

Out of the 411 cancer patients who reported post-treatment skin reactions (severity⩾1) in the nationwide Symptom Inventory, 156 (38%) received chemotherapy, 138 (34%) received radiation therapy, and 117 (28%) received both chemotherapy and radiation therapy. Histograms of the severity of skin reactions showed that the trends for patients receiving either chemotherapy or radiation therapy skewed to the right, demonstrating that more patients reported mild skin reactions than severe reactions. Interestingly, there was a more symmetric distribution of reported skin reactions in patients receiving both chemotherapy and radiation therapy. Overall, the mean severity (4.17, 4.2, and 4.6, respectively) for skin problems in each treatment group showed minimal variation (Figure 1). A non-parametric Kruskal Wallis test (*P*=0.586) and median test (*P*=0.2) did not show any significant difference in the skin problems by treatment group. Since the histograms portrayed a different distribution of skin reactions reported by patients receiving both types of treatment compared to patients receiving only chemotherapy or only radiation therapy, descriptive statistics and independent *t*-test analyses were performed to determine if there was a difference in the number of moderate (severity=3–6) or severe skin (severity⩾7) skin problems reported by each treatment group. No variation was found in the proportion of moderate skin reactions (chemotherapy=44%, radiation therapy=43%, both=42%) or in severe skin reactions (chemotherapy=21%, radiation therapy=25%, both=27%). Patients receiving both chemotherapy and radiation therapy did not report more severe skin reactions than patients receiving only radiation therapy (*P*=0.657) or chemotherapy (*P*=0.314).

### Pretreatment expectations did not influence patient report of post-treatment skin reaction

**Figure 2:**
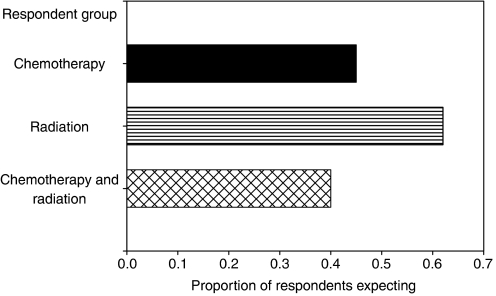
Cancer patients receiving radiation had the strongest expectations of post-treatment skin problems. A greater proportion of radiation patients (62%) reported moderate to strong certainty (scale⩾3) of having post-treatment skin problems compared to patients receiving chemotherapy (40%) or both treatments (45%). These differences were statistically significant (radiation *vs* chemotherapy, *P*=0.001; radiation *vs* both, *P*=0.003) by one-way between-groups ANOVA with Bonferroni correction.

The nationwide Symptom Inventory included a ‘pretreatment expectation section’ in which the patient was asked to indicate which of the 12 symptoms they expected to have as a result of cancer treatment. The patients were asked to rate their certainty of side effect occurrence on a five-point horizontal scale anchored by 1=‘I am certain I will NOT have this’ to 5=‘I am certain I WILL have this.’ Of the 407 patients who completed this section of the nationwide Symptom Inventory, 155 (38%) received chemotherapy, 135 (33%) received radiation therapy, and 117 (29%) received both chemotherapy and radiation therapy. One-way between-group ANOVA, with Bonferroni correction, showed significantly more patients receiving radiation therapy had stronger expectations (⩾3) of post-treatment skin problems (62%) than patients receiving chemotherapy (40%, *P*=0.001) or both treatments (45%, *P*=0.003) (Figure 2). However, pretreatment expectations did not correlate with the reported skin problems post-treatment for white (*r*=0.014, *P*=0.781) or black patients (*r*=0.021, *P*=0.936). Furthermore, a two-tailed independent *t*-test showed no significant difference in pretreatment expectations of post-treatment skin problems between black and white patients (*P*=0.32).

### Diagnosis did not significantly correlate with post-treatment skin reactions in black or white patients in the nationwide and local Symptom Inventories

**Figure 3:**
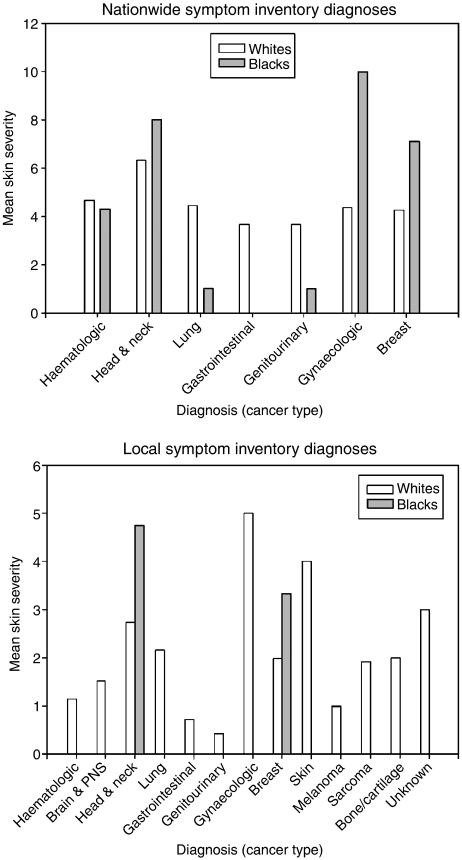
Mean skin severity by cancer diagnoses for patients in the nationwide and local Symptom Inventories. In the nationwide Symptom Inventory, the highest mean skin scores were observed for head and neck, haematologic, gynaecologic, and breast cancers for both black and white patients. For these four diagnoses, the mean skin severity was higher for black than white patients, except for haematologic cancers. In the local Symptom Inventory, the mean skin severity was higher for black than white patients for breast and head and neck cancers. Overall diagnosis did not significantly contribute to the report of post-treatment skin reactions by black or white patients.

The nationwide and local Symptom Inventories involved patients with various types of cancer. Therefore, it was necessary to determine whether or not diagnosis influenced the report and severity of post-treatment skin reactions. Figure 3 shows the mean severity of reported post-treatment skin reactions for each diagnosis on the nationwide and local Symptom Inventories. The majority of white patients (*N*=393) in the nationwide Symptom Inventory who reported a post-treatment skin reaction were diagnosed with breast (59.8%), lung (10.4%), or genitourinary (9.4%) cancers. The majority of black patients (*N*=18) in the nationwide Symptom Inventory who reported a post-treatment skin reaction were diagnosed with breast (50%), genitourinary (16.7%), or haematologic (16.7%) cancers. The mean skin reaction severity reported for lung and genitourinary cancers was higher in white (4.46 and 3.68) than black patients (1.00 and 1.00). In contrast, the mean skin reaction severity reported for breast, head and neck, and gynaecologic cancers was higher in black (7.11, 8.00, and 10.00) than white patients (4.26, 6.33, and 4.37). Bivariate correlation analysis of the nationwide Symptom Inventory did not reveal significant relationships between self-reported skin reactions and diagnosis for white (*r*=−0.38, *P*=0.456) or black patients (*r*=0.314, *P*=0.205). Additionally, linear regression analysis revealed that diagnosis did not strongly contribute to post-treatment skin reaction reported by white (SB=−0.038, *t*=−0.747, *P*=0.456) or black patients (SB=0.314, *t*=1.323, *P*=0.205).

The distribution of diagnoses in the local Symptom Inventory differed from the nationwide Symptom Inventory. The majority of white patients (*N*=166) in the local Symptom Inventory who reported a post-treatment skin reaction were diagnosed with breast (49%), lung (12.4%), and brain and PNS (11.8%) cancers. In contrast, black patients (*N*=5) in the local Symptom Inventory who reported a post-treatment skin reaction were diagnosed with head and neck (60%) or breast (40%) cancer. The mean skin reaction severity reported for head and neck and breast cancers was higher in black (6.33 and 5.00) than white patients (3.73 and 2.77). Similar to the nationwide Symptom Inventory, self-reported skin reactions did not significantly correlate with diagnosis in black (*r*=−0.506, *P*=0.326) or white patients (*r*=−0.28, *P*=0.726). Furthermore, linear regression analysis confirmed that the diagnosis did not contribute to the report of post-treatment skin reactions in black (SB=−0.56, *t*=−1.171, *P*=0.326) or white patients (SB=−0.28, *t*=−0.351, *P*=0.726). Overall, black patients were more apt to report more severe post-treatment skin problems than white patients, regardless of diagnosis.

### Black patients reported more severe skin problems in nationwide and local Symptom Inventory

**Figure 4:**
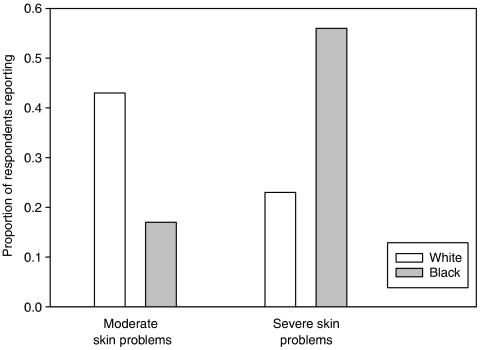
Black patients report more severe post-treatment skin reactions than white patients in nationwide Symptom Inventory. A total of 393 white and 18 black patients completed the nationwide Symptom Inventory. White (43%) patients reported more moderate skin problems (scale=3–6) than black (17%) patients. However, black (56%) patients reported more severe skin problems (scale⩾7) than white (23%) patients. The difference between black and white patients in the reporting of sever skin problems was statistically significant (*P*=0.001).

To determine if dark skin pigmentation was protective against cancer treatment-related skin problems, comparative analysis was made between the severity of skin reactions reported by white (*n*=393) and black patients (*n*=18) in the nationwide sample. Despite a greater increase in mean skin problem score (5.5 *vs* 4.2) and standard deviation (3.45 *vs* 2.7) in black compared to white patients, the non-parametric Mann–Whitney test showed no significant difference in the overall reporting of skin problems (*P*=0.152). Histograms portrayed the distribution of skin problems for white patients as skewed to the right, whereas for black patients it was U-shaped, suggesting that black patients tend to report skin reactions at both extremes (i.e., mild or severe). In concordance with this finding, independent *t*-test analysis showed a statistically significant difference (*P*=0.001) in the proportion of white and black patients who reported severe skin reactions (severity⩾7). Interestingly, severe skin problems (severity⩾7) were reported by 56% of black patients, but only 23% of white patients (Figure 4).

**Figure 5:**
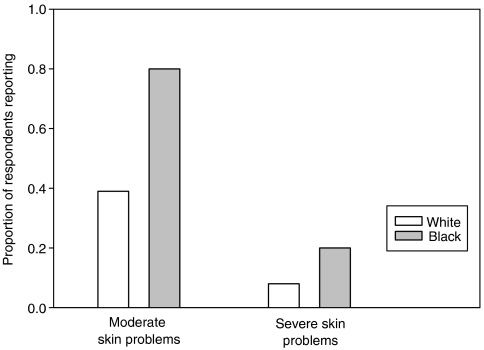
Black patients reported more post-treatment skin reactions than white patients in local Symptom Inventory. A total of 156 white and 8 black patients completed the local Symptom Inventory. Black patients reported more moderate (80%) and severe (20%) skin reactions compared to white patients (39 and 8%, respectively). Despite the small sample size, these data support the results from the nationwide Symptom Inventory.

In order to determine the consistency of the associations defined by the nationwide Symptom Inventory, statistical analysis was performed on the severity of skin reactions reported by white (*n*=161) and black patients (*n*=5) in the local weekly Symptom Inventory. Independent *t*-test analysis showed a statistically significant difference (*P*=0.006) between mean skin problem score reported by white (mean=2.98) and black patients (mean=5.8). A higher proportion of black patients reported both moderate (80%) and severe (20%) skin problems than white patients (39 and 8%, respectively) (Figure 5).

### Direct correlation between severity of skin problems and pain at treatment site

On the local Symptom Inventory questionnaire, the cancer patients were asked to rate their skin problems and pain at the site of treatment, as opposed to rating any generalised skin problems or pain, which was asked in the nationwide Symptom Inventory. Overall, the severity of skin problems at treatment site reported after 5 weeks of radiation therapy directly correlated with the severity of pain at the treatment site by Pearson's correlation (*r*=0.541, *P*<0.001). However, when correlative analysis was performed based on race, the severity of pain at treatment site did not significantly correlate with severity of skin problems at treatment site in black patients (*r*=−0.645, *P*=0.355). Regression analysis using one-way ANOVA showed that the report of pain at treatment site is a potential predictor of the report of skin problems at treatment site in white (SB=0.513, *t*=7.533, *P*<0.001), but not black patients (SB=−0.369, *t*=−0.687, *P*=0.541). Additionally, we examined the influence of total radiation exposure (total Gy) on the report of skin problems at the treatment site. No correlation was observed between total radiation dose and reported skin problems at treatment sites by white (*r*=−0.043, *P*=0.602) or black patients (*r*=−0.089, *P*=0.911). Regression analysis by one-way ANOVA confirmed that total radiation dose is not a potential predictor of reported skin problems at the treatment site for white (SB=−0.043, *t*=−0.523, *P*=0.602) or black patients (SB=−0.089, *t*=0.126, *P*=0.911).

## DISCUSSION

Radiation therapy and chemotherapy, separately or combined, are used worldwide to treat various types of cancer. Despite advances in medical technology, cancer patients experience various treatment-related ailments. For many years, it has been known that UV radiation from sun exposure is a major cause of skin cancer (Eide and Weinstock, 2005). Radiation therapy, which uses ionising radiation, and chemotherapy use toxic agents that damage tissues and DNA (Eide and Weinstock, 2005; Lopez *et al*, 2005), often resulting in nausea, depression, fatigue, and impaired cognitive functioning (Hickok *et al*, 2005; Knobf and Sun, 2005). It was not surprising that cancer patients undergoing radiation therapy expect and experience skin reactions because treatment is administered through the skin. Skin problems are one of the most frequently reported side effects of cancer treatment. This comparative study provided insight into how skin pigmentation and pretreatment expectations influence post-treatment skin reactions reported by cancer patients.

Adverse skin reactions are commonly observed with various types of cancer treatment (Alley *et al*, 2002). Common dermatologic manifestations from chemotherapy include alopecia, hyperpigmentation, hypopigmentation, erythema, and atrophy (Alley *et al*, 2002). Radiation-induced skin reactions range from mild erythema to moist desquamation and necrosis. Additionally, some chemotherapeutic drugs sensitise the skin to radiation (Alley *et al*, 2002). Our results showed no difference in the severity of skin reactions by treatment type (radiation therapy, chemotherapy, or both). However, black and white patients, regardless of the pretreatment expectations or diagnosis, differ in their perceptions of skin reactions and pain induced by cancer treatment. Although psychological factors are thought to play a role in disease burden and treatment response (Morse *et al*, 2003; Olver *et al*, 2005), our study did not demonstrate any relationship between pretreatment expectations and the report of post-treatment skin problems. Cancer patients receiving only radiation therapy expected more skin problems than cancer patients receiving chemotherapy or combination therapy.

Radiation-induced skin reactions are often unpredictable and vary by individual (Johansson *et al*, 2002; Lopez *et al*, 2005). Melanin, found in human skin, is considered to be protective against UV and ionising radiation (Nielsen *et al*, 2006). Darkly pigmented skin contains more melanin than lightly pigmented skin. Melanin is an excellent absorber of UV radiation, and its concentration in the skin is strongly affected by UV radiation (Nielsen *et al*, 2006). Untanned skin predominantly contains melanin in the basal layer of the epidermis, whereas tanned skin contains melanin throughout the epidermis. Melanin acts to remove free radicals and reactive oxygen species that are generated in skin by UV radiation (Hoogduijn *et al*, 2004). Melanocytes, the cells that synthesise melanin, are more susceptible to the damaging effects of oxidative stress than other cell types in the skin, such as keratinocytes and fibroblasts (Hoogduijn *et al*, 2004). Repeated exposure to UV or ionising radiation can disrupt melanin production and result in irreversible skin damage. Even though melanin can protect melanocytes and keratinocytes from oxidative DNA damage, *in vitro* experiments have demonstrated that the presence of reactive oxygen species during melanin synthesis can increase DNA damage (Hoogduijn *et al*, 2004). Therefore, darkly pigmented skin may be more susceptible to DNA damage from radiation due to the higher rate of melanin synthesis compared to lightly pigmented skin. Likewise, severe ionising radiation-induced skin reactions are often observed in sun-exposed regions of the skin (Harper *et al*, 2004). Besides skin pigmentation, radiation-induced skin injury is also influenced by treatment area on the body (Stone *et al*, 2003). The head and neck region, as well as the thorax region, are more prone to skin damage from radiation than the pelvic region of the body (Stone *et al*, 2003). In this study, the black patients who reported post-treatment skin reactions were diagnosed with head and neck cancer (60%) and breast cancer (40%). Although diagnosis did not significantly correlate with the report of post-treatment skin reactions for white or black patients, the statistically significant difference in mean severity of skin reaction between the two racial backgrounds could be due to the dissimilar distribution of diagnoses. Future studies involving a larger population of black cancer patients would clarify these findings.

In this study, black patients reported more severe post-treatment skin reactions than white patients. Furthermore, the total radiation exposure directly correlated with the severity of skin reactions reported by black patients, but not by white patients. Therefore, the high melanin content of darkly pigmented skin did not appear protective. It is possible that skin reactions are not visualised in darkly pigmented skin until damage is more severe. For example, pinching of skin leaves a more easily visible red mark on fair-skinned individuals, because pigment interferes with the visualisation of redness in dark-skinned individuals. Additionally, radiation-induced disturbance of skin pigmentation would be more noticeable in more darkly pigmented skin.

Over the past few years, the influence of race (ancestry and physical characteristics) and ethnicity (behavioural and cultural distinctions) on the experience of pain has become a growing area of research (Edwards *et al*, 2001a; Morse *et al*, 2003). Many studies have demonstrated that black patients report higher levels of clinical pain and greater pain-related disability than white patients (Edwards *et al*, 2001a; Campbell *et al*, 2005). Additionally, racial differences in pain have been found to vary by anatomical site. For instance, black patients tend to report more pain associated with glaucoma, arthritis, orofacial injury, and migraine headaches (Riley *et al*, 2002; Campbell *et al*, 2005). Riley *et al* (2002) and Sanders *et al* (1992) showed that given the same pain stimulus, black patients had a stronger perception and response to the pain stimulus than white patients. Our study supported these previous findings by showing a direct correlation between the severity of self-reported pain and skin reaction at the treatment site in black patients, but not in white patients.

The therapeutic effects of ionising radiation are based on the greater capacity of normal tissues to repair DNA damage compared to rapidly proliferating tumour cells. Unfortunately, radiation treatment for cancer must be administered through the skin, (Harper *et al*, 2004). Even though skin is not the target of treatment, it is damaged during the treatment process. Radiation-induced skin injury is influenced by treatment-related factors, such as volume of treatment area and fraction dose size, and by patient-related factors (Harper *et al*, 2004). For instance, more severe radiation-induced skin problems occur in large-breasted women and obese individuals. Other patient-related risk factors for skin problems from cancer treatment include age, smoking status, infection of surgical wounds, and genetics (Harper *et al*, 2004). Mutations in the ataxia-telangiectasia (*ATM*) gene have been associated with increased radiosensitivity and radiation-induced morbidity (Iannuzzi *et al*, 2002; Harper *et al*, 2004). *ATM* heterozygosity occurs in approximately 1% of the general population and predicts subcutaneous late responses to radiation therapy, but not acute effects (Iannuzzi *et al*, 2002). Therefore, patients containing a mutated *ATM* gene might be predisposed to late radiation-induced skin injury. It is also known that point mutations and deletions in mitochondrial DNA (mtDNA) increase cellular radiosensitivity (Prithivirajsingh *et al*, 2004). Unlike nuclear DNA, mtDNA lacks an efficient DNA repair mechanism and is more vulnerable to the accumulation of DNA damage (Prithivirajsingh *et al*, 2004; Wilding *et al*, 2006). High levels of mtDNA mutations in the skin could predispose cancer patients to radiation-induced skin problems. Unfortunately, we were unable to correlate the Symptom Inventory data with genetic alterations because the collection of blood and tissue samples from patients was not performed in this observational study based on clinical data. Next steps in understanding the differences observed in this study would include assessing the relationship of our findings to these known contributors to radiation sensitivity. These explanations for the difference in the severity of post-treatment skin reactions reported by black and white patients should be explored in a future clinical study.

Although this comparative study was able to demonstrate statistically significant racial differences in the severity of post-treatment skin reactions and pain reported by cancer patients, black subjects were underrepresented in both surveys in comparison to white subjects. The small sample size of black patients could have biased the statistical results, especially if these individuals represent extremes of the general population. Further clinical studies should include a larger sample population of black patients to determine more precisely the distribution of reported post-treatment skin reactions and pain in black and white patients. Additionally, a larger sample size would increase the statistical power and validity of the results, as well as the ability to understand specific consequences of treatments such as anti-epidermal growth factor receptor therapy, which commonly cause an acne-like rash (Sipples, 2006). Additionally, future studies would benefit from extensive clinical data, such as disease stage, area of treatment, documentation of skin problems, and blood samples to examine predisposing genetic factors. Our study did show similar trends in two separate surveys (national and local); both suggesting that black and white patients differ in their perception of skin reactions and pain induced by cancer treatment. Further elucidation of the role of skin pigmentation and the influence of race and ethnicity on skin reactions and pain experienced by cancer patients could improve cancer treatment symptom management for future patients.

## Figures and Tables

**Table 1:** Demographics for cancer patients

	**Nationwide Symptom Inventory (*n*=656)**	**Local Symptom Inventory (*n*=308)**
*Overall patient demographics*/		
*Age (years)*		
<61	320 (49%)	159 (52%)
⩾61	336 (51%)	149 (48%)
		
*Sex*		
Female	429 (65%)	167 (54%)
Male	227 (35%)	141 (46%)
		
*Race*		
White	623 (95%)	298 (97%)
Black	33 (5%)	10 (3%)
		
	**Nationwide Symptom Inventory (*n*=411)**	**Local Symptom Inventory (*n*=166)**
*Demographics for patients reporting post-treatment skin reactions*		
*Age (years)*		
<61	220 (54%)	111 (67%)
⩾61	191 (46%)	55 (33%)
		
*Sex*		
Female	299 (73%)	113 (68%)
Male	112 (27%)	53 (32%)
		
*Race*		
White	393 (96%)	161 (97%)
Black	18 (4%)	5 (3%)

**Table 2:** Cancer diagnoses for patients in nationwide and local Symptom Inventory

	**White**		**Black**	
**Type of cancer**	**All patients**	**Patients reporting post-treatment skin reaction**	**All patients**	**Patients reporting post-treatment skin reaction**
*Nationwide Symptom Inventory*				
Haematologic	37	24	4	3
Head and neck	8	6	1	1
Lung	62	41	1	1
Gastrointestinal	44	31	0	0
Genitourinary	117	37	9	3
Gynaecologic	32	19	2	1
Breast	322	235	16	9
Unknown primary	1	0	0	0
Total	623	393	33	18
				
*Local Symptom Inventory*				
Haematologic	7	3	0	0
Brain and PNS	37	19	2	0
Head and neck	15	11	4	3
Lung	32	20	0	0
Gastrointestinal	17	6	1	0
Genitourinary	59	10	0	0
Gynaecologic	1	1	0	0
Breast	110	79	3	2
Skin	3	2	0	0
Melanoma	2	1	0	0
Soft tissue Sarcoma	12	7	0	0
Bone or cartilage	2	1	0	0
Unknown primary	1	1	0	0
Total	298	161	10	5

PNS=peripheral nervous system.
